# N-Acetyl Cysteine Restores the Diminished Activity of the Antioxidant Enzymatic System Caused by SARS-CoV-2 Infection: Preliminary Findings

**DOI:** 10.3390/ph16040591

**Published:** 2023-04-14

**Authors:** María Elena Soto, Linaloe Manzano-Pech, Adrían Palacios-Chavarría, Rafael Ricardo Valdez-Vázquez, Verónica Guarner-Lans, Israel Pérez-Torres

**Affiliations:** 1 Department of Immunology, Instituto Nacional de Cardiología Ignacio Chávez, Juan Badiano 1, Sección XVI, Tlalpan, Mexico City 14080, Mexico; 2 Department of Cardiovascular Biomedicine, Instituto Nacional de Cardiología Ignacio Chávez, Mexico City 14080, Mexico; 3 Critical Care Unit of the Temporal COVID-19 Unit, Citibanamex Center, Mexico City 11200, Mexico; 4 Department of Physiology, Instituto Nacional de Cardiología Ignacio Chávez, Juan Badiano 1, Sección XVI, Tlalpan, Mexico City 14080, Mexico

**Keywords:** SARS-CoV-2, N-acetyl cysteine, antioxidant enzymatic system, COVID-19, total antioxidant capacity

## Abstract

SARS-CoV-2 infects type II pneumocytes and disrupts redox homeostasis by overproducing reactive oxygen species (ROS). N-acetyl cysteine (NAC) is a precursor of the synthesis of glutathione (GSH) and it restores the loss of redox homeostasis associated to viral infections. The aim of the study is to evaluate the effect of the treatment with NAC on the enzymatic antioxidant system in serum from patients infected by SARS-CoV-2. We evaluated the enzymatic activities of thioredoxin reductase (TrxR), glutathione peroxidase (GPx), -S-transferase (GST), and reductase (GR) by spectrophotometry and the concentrations of the glutathione (GSH), total antioxidant capacity (TAC), thiols, nitrites (NO_2_^–^), and lipid peroxidation (LPO) in serum. The activity of the extracellular super oxide dismutase (ecSOD) was determined by native polyacrylamide gels, and 3-nitrotyrosine (3-NT) was measured by ELISA. A decrease in the activities of the ecSOD, TrxR, GPx, GST GR, (*p* = 0 ≤ 0.1), and the GSH, TAC, thiols, and NO_2_^–^ (*p* ≤ 0.001) concentrations and an increase in LPO and 3-NT (*p* = 0.001) concentrations were found in COVID-19 patients vs. healthy subjects. The treatment with NAC as an adjuvant therapy may contribute to a reduction in the OS associated to the infection by SARS-CoV-2 through the generation of GSH. GSH promotes the metabolic pathways that depend on it, thus contributing to an increase in TAC and to restore redox homeostasis.

## 1. Introduction

According to the WHO Coronavirus (COVID-19) Dashboard, there have been 761,402,282 confirmed cases of the disease worldwide until April 2023, and 6,887,000 deaths by the new type 2 coronavirus (SARS-CoV-2), that causes severe acute respiratory syndrome. Moreover, 13,317,121,247 vaccine doses have been applied worldwide. SARS-CoV-2 type 2 infects the pneumocytes in the alveoli of the lungs, by the attaching its S protein with the angiotensin-converting enzyme 2 (ACE2) receptor and the serine protease TMPRSS2, facilitating the fusion of the viral membrane with the pneumocyte cellular membrane [1]. The structural and non-structural (nsp) proteins of SARS-CoV-2 possess the genetic information needed to evade the immune response strategies of the host and to sequester the cellular machinery to favor replication [2]. After evading the immune response, the virus enters the cell by endocytosis and progressively triggers a cytokine storm that results in systemic hyper inflammation caused by the immunologic system of the host. This storm is aimed at facing and fighting the infection [3]. On the other hand, the individuals with pathologies such as cardiovascular disease, chronic obstructive pulmonary disorder, asthma, diabetes, insulin resistance, overweight, obesity, immune deficiency, chronic renal impairment, neurodegenerative diseases [4], and old age are more susceptible to infection since they undergo alterations in the immunologic and metabolic systems in which the oxidative background is elevated facilitating the entrance of the SARS-CoV-2 [3]. In these conditions, the redox homeostasis is altered leading to an overproduction of the reactive oxygen species (ROS) through the activity of NADPH oxidases, inducible nitric oxide synthase (iNOS), xanthine oxidase, and the uncoupling of the mitochondrial transport chain. This contributes to a positive feedback loop between the inflammatory state and oxidative stress (OS) that may lead the patient to a moderate or severe state with multiple organ failure and possibly a fatal outcome [5].

Studies in COVID-19 patients have described the infection by the SARS-CoV-2 virus courses with OS and nitrosative stress (NSS) where the tripeptide-thiol glutathione (GSH) is seriously compromised, and its concentration is diminished resulting in increased lipid peroxidation (LPO) and decreased total antioxidant capacity (TAC) [6]. The decrease in GSH also contributes to reducing the activities of enzymes that employ it, such as the thioredoxin reductase (TrxR), glutathione peroxidase (GPx), glutathione-S-transferase (GST), glutaredoxin and glutathione reductase (GR). The alteration in these enzymes causes an imbalance in the antioxidant enzymatic system that could drive to apoptosis in lymphocytes and reduce the percentages of basophils, eosinophils, and monocytes [7].

In contrast, key enzymes that do not employ GSH but participate in the detoxification processes of the anion superoxide (O_2_^–^) are also present and they include the superoxide dismutase (SOD) isoforms [8]. The activity of these enzymes is altered in the SARS-CoV-2 infection and contributes to the loss of the redox homeostasis. These enzymes have three isoforms, SOD-1 or mitochondrial, SOD-2 or cytosolic, and SOD-3 or extracellular, which are present in the serum [9]. These enzymes have metalloids in their catalytic center such as manganese, zinc, and copper and act as the first line of defense against the increase in ROS. They are of vital importance because of their enormous turnover capacity in the presence of large concentrations of the substrate, which is higher than that of other antioxidant enzymes and whose deficiency can cause death [10].

On the other hand, to be able to counteract the SARS-CoV-2 infection, a combined therapy aiming at two directions would be desirable: (A) prevention and (B) treatment. The latter one is the most effective measure to stop the disease. Although, at present, various vaccines and antiviral agents against SARS-CoV-2 are available, the search for adjuvant therapies that can contribute to reducing the severity of the infection through clinical and basic studies continues. With respect to treatment, it has been divided into two phases. The first phase is aimed at lowering the viral load that is the source and origin of the chronic inflammation and the cytokines storm. In this sense, several antiviral molecules have been authorized in various countries: Remdesivir, favipiravir, umifenovir, molnupiravir, and the combination of nirmatrelvir–ritonavir [11,12]. The second phase restores the loss of the redox homeostasis that causes the cytokine storm and inflammation. The use of antioxidants might decrease the exacerbated inflammatory response without collateral effects. The two phases should be aimed at simultaneously, and the therapeutic strategy should be selected avoiding subsequent collateral damage to the patients. In this sense, several studies have used drugs and natural antioxidant substances to reduce OS and NSS in patients infected by SARS-CoV-2 [3].

N-acetyl cysteine (NAC), a precursor for the synthesis of GSH, possesses mucolytic properties and has a broad range of antioxidant and anti-inflammatory activities [13]. The effect of NAC is exerted by deacetylation to yield L-cysteine which is the rate-limiting amino acid in the synthesis of GSH. An increase in this amino acid induces the activity of the glutamate cysteine ligase (GCL), a key enzyme in the synthesis of GSH [14]. Furthermore, GSH may interact with hydrogen sulfide whose levels are low in acute respiratory distress syndrome (ARDS) and chronic obstructive pulmonary disease (COPD). These conditions are associated with the SARS-CoV-2 infection. In this sense, NAC can exogenously increase H_2_ S by a synergistic effect with GSH and protect from the damage in the lungs of the patients infected by SARS-CoV-2 [15]. Therefore, the aim of this study is to evaluate the effect of treatment with NAC before and after on some enzymes of the enzymatic antioxidant system in patients infected by SARS-CoV-2.

## 2. Results

### 2.1. Demographic Characteristics of the Patients Infected by SARS-CoV-2

A total of 16 patients were included, 13 (77%) were men and 3 (23%) were women. The demographic characteristics are shown in Table 1. Patients had a median age of 55 with 27–84 min-max range. The median body mass index was 28 with 23–38 min-max range kg/m^2^. Normal weight was found in 6 (35%), overweight in 7 (41%), and obesity in 3 (24%). Comorbid conditions prior to SARS-CoV-2 infection were dyslipidemia in 7 (41%), systemic arterial hypertension in 8 (47%), and diabetes mellitus in 8 (47%). The temperature median was of 36 °C with 36.5–37.2 min-max range. Other variables expressed as median and minimum–maximum ranges, respectively, that are included are: arterial blood oxygen pressure (PaO_2_, 70, 59–179 mmHg), partial pressure of carbon dioxide (PCO_2_, 31.7, 12.2–39 mmHg), Kirby’s index which is PaCO_2_/inspired fraction of oxygen (FiO_2_ 131.5, 45–282 mmHg), oxygen saturation (SpO_2_/FiO_2_, 119, 61–290 mmHg), Heart rate Ipm (74, 20–116), the arterial pressure (78, 71–102 mmHg), the body temperature (36, 36.5–37.2 °C), total cholesterol (1363, 89–210 mg/dL), triglycerides (186.5, 80–726 mg/dL), high-density lipoprotein (26, 14–40 mg/dL), low-density lipoprotein (62, 40–110 mg/dL), leukocytes (8.8, 3.5–14.9 10^3^/μL), lymphocytes (8.3, 0.14–1.54 10^3^/μL), platelets median (253, 164–408 10^3^/μL), ferritin (435.4, 146–2592 ng/mL), D-dimer (605, 200–2100 μg/mL), CRP (143.15, 44.7–221 mg/L), IL-6 (16, 7.8–324 pg/mL), NR (8.4, 6–67), procalcitonin (0.23, 0.08–11.4 ng/mL). The sepsis score according to SOFA index was (0, 0–3), APACHE (5.5, 4–8), and SAPS (27, 18–32). The patients with moderate and severe condition were 8, respectively (50/50%), and the number of days with mechanic ventilation was (13, 5–28), days in ICU were (17, 3–30), and death 2 (12%).

### 2.2. Demographic Characteristics of the Healthy Subjects

A total of 20 healthy subjects (HS) were included in this study, of which 10 (50%) were men and 10 (50%) were women. HS had a median age of 53 min 30 and max 63 ranges. The values of glucose, insulin, cholesterol, triglycerides, HDL, LDL, and creatinine are shown in Table 2. In these subjects, there was no suspicion of degenerative disorders, inflammatory disease, and/or autoimmune diseases. Renal failure, hypertension, cardiovascular disease, diabetes mellitus, and dyslipidemia were not present. Table 2 shows the biochemical characteristics of the HS.

### 2.3. Oxidative Stress Markers

TAC showed a significant decrease in the patients infected by SARS-CoV-2 before the NAC treatment in comparison with HS and after the treatment (*p* < 0.001). The GSH levels showed the same tendency as TAC with a significant decrease in the before the NAC treatment patients (*p* < 0.001) versus HS and after the NAC treatment. The total thiol groups was decreased in the patients infected by SARS-CoV-2 before the NAC treatment vs. HS (*p* < 0.001) but the treatment with NAC increased them, showing statistically significant changes (*p* ≤ 0.05). However, the median of LPO index showed a significant increase in the patients infected by SARS-CoV-2 before the NAC treatment (*p* = 0.01 and *p* ≤ 0.05) in comparison with HS and in the patients after the treatment. The NO_2_^–^ levels in the patients before the NAC treatment showed a significant decrease (*p* < 0.001) in comparison to HS and after the NAC treatment. Finally, the 3-NT concentration was significantly increased in the patients infected by SARS-CoV-2 before the NAC treatment with respect to HS (*p* < 0.001); however, the NAC treatment caused a significant decrease in these patients (*p* ≤ 0.05). The OS markers are shown in Table 3.

### 2.4. Enzymatic Activities the ecSOD, GPx, TxrR, GST, GR, and ALDH

**Figure 1:**
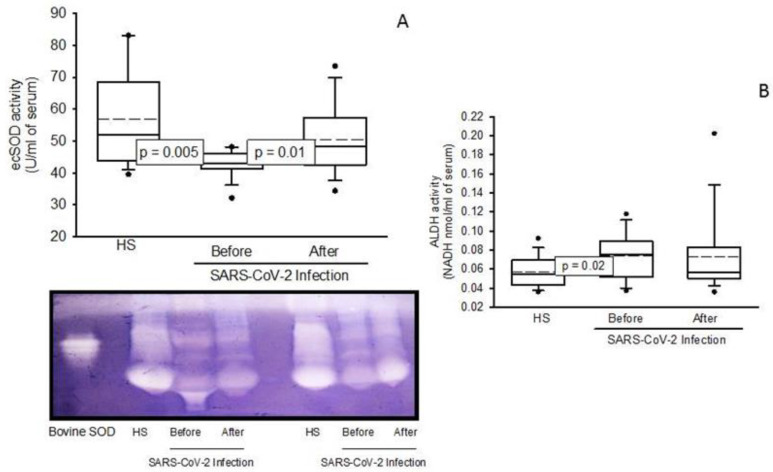
Activities of the ecSOD (**A**) and ALDH (**B**) in the HS and the patients infected by SARS-CoV-2 before and after the NAC treatment. In the panel, a representative gel of ecSOD activity is presented on a native 10% polyacrylamide gel. In the first lane, the activity of the commercial enzyme (bovine SOD-2) is observed, which is necessary to quantify the activity of the enzyme. The results are shown in plot boxes and whiskers in percentiles 75, 25, the median and the bridged midline, as well as outliers, since the sample size is small and is not normally distributed. Abbreviations: ecSOD; extra cellular super oxide dismutase, ALDH; aldehyde deshydrogenase, HS; healthy subjects, NAC; N-acetyl cysteine.

**Figure 2:**
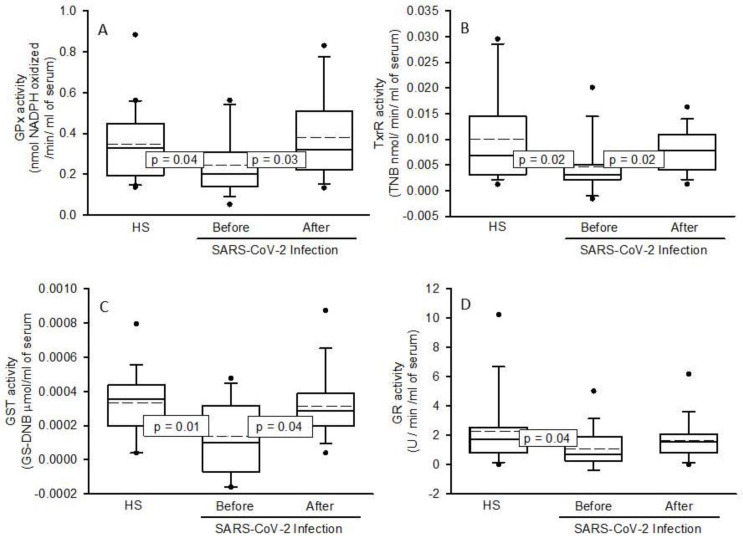
Activities of the GPx (**A**), TrxR (**B**), GST (**C**), and GR (**D**) in COVID-19 patients with the NAC treatment. The results are shown in plot boxes and whiskers in percentiles 75, 25, the median and the bridged midline, as well as outliers, since the sample size is small and is not normally distributed. Abbreviations: GPx = glutathione peroxidase, TrxR = thioredoxin reductase, GST = glutathione-S-transferase, GR = glutathione reductase, HS; healthy subjects, NAC; N-acetyl cysteine.

The activities of the antioxidant enzymes showed a significant decrease in the patients infected by SARS-CoV-2 before the NAC treatment (ecSOD; *p* = 0.005 Figure 1A, GPx; *p* = 0.04 Figure 2A, TxrR; *p* = 0.02 Figure 2B, GST; *p* = 0.01 Figure 2C and GR; *p* = 0.04 Figure 2D) in comparison with the HS. But the NAC treatment increased the activities of these enzymes in the patients infected by SARS-CoV-2 (ecSOD; *p* = 0.01 Figure 1A, GPx; *p* = 0.03 Figure 2A, TxrR; *p* = 0.02 Figure 2B, GST; *p* = 0.04 Figure 2C). However, the GR activity only showed a tendency to increase but without showing a significant change, Figure 2D. The activity of ALDH was increased in the patients infected by SARS-CoV-2 before the NAC treatment (*p* = 0.02) in comparison to HS, and the treatment with NAC did not modify it, despite a tendency to increase its activity, Figure 1B.

## 3. Discussion

NAC is a stable form of the non-essential amino acid cysteine, being a precursor of the GSH tripeptide-thiol. It increases the activities of redox enzymes that employ it including TrxR, GPx, GST, glutaredoxin, and GR. NAC also favors the activity of the enzyme GCL, which participates in the GSH synthesis [16]. It contains sulfur and acts as a stabilizer for the formation of protein structures through reduction of thiol bridges. It has mucolytic properties by breaking disulfide bonds in mucous secretions rendering them less viscous and it is currently being used in septic shock and EPOC [17]. NAC is also less expensive for the population; it has very low toxicity; and has been approved by the FDA. Diverse studies have shown that the administration of NAC by different routes (orally, inhaled or intravenously) can contribute to suppressing SARS-CoV-2 replication, decrease the infection, and strengthen the immune and antioxidant systems [18]. Potential therapeutic benefits of NAC include extracellular scavenging of free radicals, replenishing and increasing GSH, suppression of the cytokine storm, and protection of T cell, thus mitigating inflammation and tissue injury. The combination of NAC with administrations of antiviral agents could reduce the hospital admission rate, mechanical ventilation, and mortality [18].

In this sense, the aim of this study is to evaluate the effect of NAC treatment before and after on the activities of some enzymes of the enzymatic antioxidant system in patients infected by SARS-CoV-2.

Our results show that the activity of ecSOD is decreased in the patients infected by SARS-CoV-2. This isoform of the enzyme participates in the first line of detoxification against the O_2_^–^ in the blood reducing it to H_2_ O_2_. It is expressed in the sub endothelial space, in the surface of the vascular smooth muscle cells in blood vessels, and may be secreted into the extracellular space, hence found in the blood. It has a direct action on ROS, thus limiting oxidative lung injury. Our results suggest that the activity of ecSOD may be compromised in the patients infected by SARS-CoV-2 and therefore this enzyme is incapable of dismutating two O_2_^–^ to H_2_ O_2_, resulting in an increase in OS, as has been previously reported [19].

The patients infected by SARS-CoV-2 also show a deficiency in metalloids such as zinc, selenium, copper, and manganese among others. This deficiency can probably decrease the activity of some antioxidant metallo-enzymes including the SOD isoforms and seleno-enzymes such as TrxR and GPx that contribute to an increased OS [15]. The ecSOD activity may also be decreased because of the excess of substrate, despite its great replacement capacity to dismutate O_2_^–^ [20]. However, the NAC treatment increases the activity of ecSOD. This could be due to an indirect effect resulting from an increase in the GSH concentration, which lowers ROS, being an antioxidant molecule. In this same sense, the decrease in the activity of TrxR and GPx observed in the patients infected by SARS-CoV-2 can contribute to OS by abolishing detoxification of other ROS such as H_2_ O_2._ The levels of ROS are increased during hyper inflammation by SARS-CoV-2, due to the activity of the innate immune system to fight viral infection through the peroxidases family [21]. H_2_ O_2_ is the substrate for the isoforms of GPx and TrxR. However, the high concentration of this ROS may, in turn, lower the activities of GPx and TrxR. Therefore, our results show that a decrease in the activities of these enzymes could be due to an excessive increase in H_2_ O_2_ in the SARS-CoV-2 infection [21,22].

We also found that GSH was depleted in the patients infected by SARS-CoV-2 in this study, as has been previously reported [15]. In addition, the protease M^pro^, a cysteine protease of the SARS-CoV-2, which is encoded by the nsp5 virus, can also interact with GPx1 and with other seleno-enzymes such as TxrR1 and favor the abolition of their activities increasing OS. Other nsp proteins such as the 12 and 13 can interact with GCL and decrease the synthesis of GSH [22]. Furthermore, an increase in the TrxR activity could block the entrance of the virus via a reduction of the ACE2 receptor through decreased thioredoxin and other thiols groups [23]. In this sense, the ACE2 receptor of the host has three disulphide bonds through conserved cysteines that are required in an oxidative environment. The presence of these bonds renders thiols rusty and allows for the infiltration of the SARS-CoV-2 virus into the cells. However, disulfide bridges are mainly reduced by the thioredoxin system, and the decrease in the TrxR activity diminishes the thiol groups as shown in our results, thus favoring the entrance of the virus [23].

The antioxidant therapy with the NAC increases the activities of GPx, TrxR, and thiol groups and this could contribute to a decrease in OS and the severity of the disease by interfering with the entrance of the virus into the host cells. TrxR possesses a seleno-cysteine in its catalytic site and NAC may supply this amino acid. The virus may also decrease TrxR and the glutaredoxin systems, which could be a viral strategy to inhibit DNA synthesis. A reduction in the synthesis of DNA may increase the pool of ribonucleotides for RNA synthesis, thereby enhancing virion production, a process which is unsustainable without the participation of the TrxR and glutaredoxin systems [23,24].

On the other hand, few studies have paid attention to GR in SARS-CoV-2 infection. This enzyme is essential for the GSH regeneration. A study in 104 subjects showed that the activity of GR was decreased in the SARS-CoV-2 infection [25]. Our results show a decrease in the activity of this enzyme and, although there was a tendency of the activity of GR to increase due to treatment with NAC, it did not show a statistical difference. This suggests that the SARS-CoV-2 infection decreases the activity of this enzyme and may lead to an increase in GSSG and a decrease in GSH, which would contribute to the positive feedback process between the inflammatory process and OS. The reduction in the activity of GR could lead to neutrophil dysfunction during the infectious processes as has been previously reported in GR-deficient mice and humans [26]. The decreased activity of this enzyme in neutrophils mediates S-glutathionylation of the key protein NOX in vitro and in vivo [27]. The S-glutathionylation process regulates the GHS conjugation with a cysteine thiol group on target proteins and this may modulate their function. The GR activity is also essential to promote the innate immune response against microorganism [28]. Therefore, the treatment with NAC could increase the activity this enzyme and favor GSH regeneration, thus contributing to decrease OS.

In the same sense, the other enzymes that participate in the S-glutathionylation process showed a decrease in their activity in this study, including GST. GST is a superfamily of isoenzymes that includes the α, μ, π, ω, θ, δ, ς, σ, κ, and m isoforms. These enzymes are multifunctional and are found in plasma, cell membranes, cytoplasm, endoplasmic reticulum, mitochondria, and the nucleus [29]. They participate as protective enzymes for exogenous and endogenous cellular detoxification and neutralization of the xenobiotics, oxidized lipids, ROS, and they decrease the rate of LPO through GSH conjugation. This conjugation increases the solubility of the toxic products, facilitating their excretion from the cell. They also play an important role in idiopathic pulmonary fibrosis and lung fibro genesis [29]. Several studies have reported that SARS-CoV-2 decrease the activity of these enzymes [30]. A study in patients with COVID-19 ranging in age from 20 to 58 years showed a decrease in the GSTp1 and there was a correlation with a higher prevalence of deaths in males in comparison with female patients [31]. Another study showed that the frequency of GSTM1−/−, GSTT1−/−, and GSTM1−/−/GSTT1−/− was higher in severe COVID-19. In patients with both GSTM1 + /+ and GSTT1−/− genotypes there was a poor survival rate. Therefore, patients with COVID-19 having the GSTT1−/− genotype had a high mortality rate [32]. However, the treatment with NAC favored an increase in this enzyme in the present study. This effect may be attributed to the increase in the GSH concentration by the treatment because it is a substrate for this enzyme, resulting in a decreased LPO. Nevertheless, the decrease in the activity of this enzyme could favor the accumulation of the LPO products such as 4-hydroxy-2-transnonenal (4-HNE) or malondialdehyde. LPO is the result of the chain reaction of OH^–^ on the unsaturated fatty acid and it is increased in the presence of transition metals such as Fe. The COVID-19 patients develop ferroptosis due to hijacking of the function of mitochondria and the knockdown of the GPx 4 isoform by the virus [33]. Furthermore, the peroxidized lipids damage the cell membrane.

However, the GST isoforms are not the only enzymes involved in reducing the formation of reactive lipid aldehydes. Other enzymes such as the ALDH superfamily are also employed for detoxification [34]. These enzymes are expressed in most human tissues, including immune cells, and are crucial for the metabolism of endogenous aldehydes, such as formaldehyde and 4-HNE which are responsible for the elimination of non-reactive products. In this sense, our results show that the activity of ALDH was increased. The role of this enzyme in SARS-CoV-2 has not been reported and few works have addressed its participation in other viral infections. However, there is an increase in this enzyme in B and C hepatitis infection [35]. The ALDH increase in SARS-CoV-2 infection could be a compensatory mechanism of protection that tries to detoxify the excess of OS associated with 4-HNE. It could also be the result of the expression of interferon to fight infections [36]. The increase would also compensate for the GST decrease.

On the other hand, a decrease in the TAC reflects the alteration of the antioxidant enzymes, GSH, thiols and an increase in the LPO, NO_2_^–^, and 3-NT. However, the treatment with NAC may restore this alteration and, as a result, the increase in the TAC and redox homeostasis tend to balance themselves [37]. This could contribute to enhance the host’s innate immune system.

Moreover, the loss of the redox homeostasis is associated with the expression of the inducible nitric oxide synthase (iNOS) and nitric oxide (NO) exacerbation that could lead to the formation of peroxynitrites (ONOO^–^). These ROS may affect enzymatic and protein functions by modifying tyrosine residues and/or thiol groups, leading to the formation of oxidized thiol groups and 3-NT. [38]. The increased formation of O_2_^–^ due to the loss of the activity of ecSOD may react with NO and decrease its concentration; however, it would increase ONOO^–^ which has a longer half-life than O_2_^–^ and is more toxic, resulting in NSS [39]. This contributes to the inflammatory process associated with the interleukin storm. In addition, the NO_2_ is diminished, but the 3-NT is increased in COVID-19 [15]. Our results agree with what was previously described. In addition, decreased NO levels may induce elevated circulating pro-inflammatory cytokines, platelet aggregation, chemokine expression, proliferation of vascular smooth muscle cell, oxidation of LDL lipoprotein, expression of vascular cell adhesion molecule-1, stimulation of thrombolysis by monocyte chemotactic protein-1. These alterations contribute to damage the lungs. Although the treatment with NAC reverses these alterations, the exact mechanism by which this effect is produced is still unknown and further studies are required for elucidation.

Perspectives: When SARS-CoV-2 attaches to the ACE2 receptor, it leads to an increase in angiotensin 2 and a decrease in angiotensin 1–7 [40]. This change increases O_2_^–^ and a positive feedback process between the OS and the inflammatory process that, in turn, causes endothelial dysfunction, favoring the increase in von Willebrand factor and causing thrombosis [41]. The NAC treatment that favors the GSH synthesis could reduce the von Willebrand factor that forms blood clots [42]. Blood clotting issues in patients infected by SARS-CoV-2 are currently being evaluated and the potential effectiveness of taking GSH alone orally and IV, rather than its precursor NAC is being tested. The alpha-lipoic acid may also represent another novel treatment approach to block NF-κB and treat the cytokine storm and respiratory distress in patients with pneumonia caused by infection with SARS-CoV-2. Therefore, NAC, GSH, and micro minerals can be an effective complement in the treatment against SARS-CoV-2 infection due to their fundamental role in reducing OS, reducing the inflammatory state, and restoring the homeostasis of blood coagulation [43,44].

**Figure 3:**
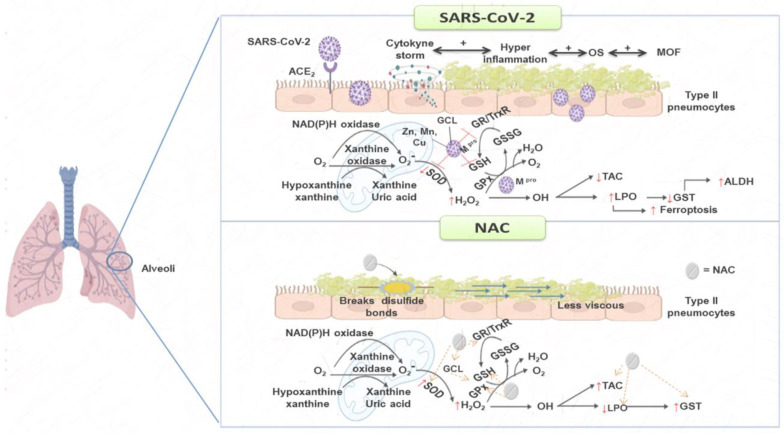
The treatment with NAC as an adjuvant therapy may contribute to reducing the OS associated with SARS-CoV-2 infection through GSH generation, which favors the increase in the enzymes that employed this antioxidant molecule. Abbreviations: Cu = copper, GCL = glutamate cysteine ligase, GPx = glutathione peroxidase, GR = glutathione reductase, GSH = glutathione, GSSG = oxidized glutathione, GS T= glutathione-S-transferase, H_2_ O_2_ = hydrogen peroxide, LPO = lipoperoxidation, Mn = manganese, MOF = multiple organ failure, NAC = N-acetylcysteine, O_2_^–^ = superoxide anion, OH = hydroxyl, OS = oxidative stress, SOD = superoxide dismutase, TAC = total antioxidant capacity, TrxR = thioredoxin reductase, Zn = zinc.

This study also confirms that the application of antioxidant therapies such as NAC treatment could help in the prevention and mitigation of the loss of redox homeostasis in the patients infected by SARS-CoV-2, and thereby have a beneficial impact by decreasing the infection and improving patient survival. These relevant findings suggest the need of conducting multicentric studies or systematic studies providing therapies with antioxidants that may improve the loss of redox homeostasis. Figure 3 summarizes the NAC treatment in the patients infected by SARS-CoV-2.

### Study Limitations

One of the limitations of this study was the sample size. The time in which the antioxidant therapy with the NAC was given, could have also been longer, but due to the lack of budget, this was not possible. However, the results obtained in this study are promising. Therefore, we suggest that the treatment with NAC should be given for the double of the time used in this study. Another limitation is the absence of a control group matched by the presence of comorbidities such as obesity, diabetes, and hypertension, which may affect the results and the conclusion.

## 4. Materials and Methods

### 4.1. Description of the Population Studied

**Figure 4:**
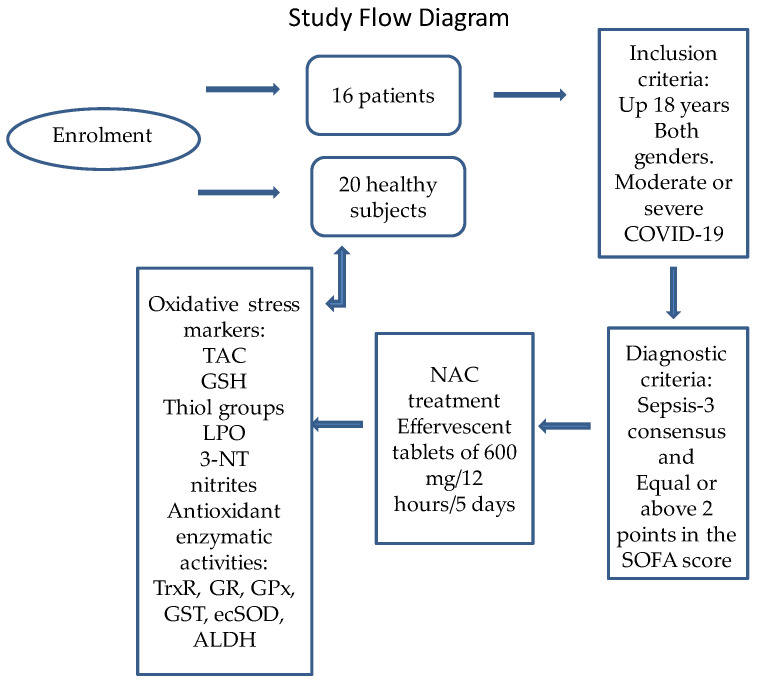
Study flow diagram. Abbreviations: GPx = glutathione peroxidase, GR = glutathione reductase, GSH = glutathione, GST = glutathione-S-transferase, LPO = lipoperoxidation, NAC = N-acetylcysteine, SOD = superoxide dismutase, TAC total antioxidant capacity, TrxR = thioredoxin reductase, 3-NT = nitrotyrosine, ALDH = aldehyde deshydrogenase activity.

This was an open, prospective, analytical, and longitudinal study (before-after treatment) in 16 patients with COVID-19 who received NAC and the results were compared with those obtained in 20 healthy subjects that acted as a control group. Inclusion criteria: The patients with COVID-19 were 18 years old or more and were admitted to the ICU of the CITIBANAMEX Center and developed or not septic shock, secondary to moderate or severe pneumonia by COVID-19. The treatment was applied between August and September of 2020. Ethical approval was obtained by the local ethics committee on 19th August 2020 (Control-9867/2020, register REG. CONBIOETICA-09-CEI-011–20160627). The protocol was registered (TRIAL REGISTRATION: ClinicalTrials.gov Identifier: NCT 04570254). Diagnostic criteria for septic shock were based in the Sepsis-3 consensus [45]. A written informed consent for enrollment or consent to use patient data was obtained from each patient or their legal surrogate, in accordance with the Helsinki declaration, as amended by the congress of Tokyo, Japan [46]. Patients considered to have septic shock had to have an acute increase of at least 2 points in the SOFA score [43], lactate levels ≥2 mmol/L, and they had to be dependent on a vasopressor for at least 2 h before the time of enrollment. To evaluate organ dysfunction, the SOFA score (neurologic, respiratory, hemodynamic, hepatic, and hematologic) was calculated at admission and during all of the days of treatment [47]. The hospitalized patients were classified as moderate or severe; this classification was decided according to their ventilatory status. Patients with the severe condition required invasive mechanical intubation according to the Berlin criteria for ARDS [48]. Exclusion criteria were: patients that were under chronic use (last 6 months) or recent use of steroids, statins, antioxidants, and NAC; patients younger than 18 years; patients that were not able to grant an informed consent, refused to be included; and also pregnant woman or breastfeeding. Figure 4 describes the study flow diagram.

In all patients, the use of hydroxychloroquine or antivirals was not considered since they received individualized management according to a proposed algorithm [3]. At the time this study was carried out, the patients were not vaccinated against SARS-CoV-2 because a vaccine had not yet been developed. Some results related to this study were previously reported by Chavarría et al. during the before treatment and after treatment evaluation with NAC [49].

### 4.2. Detection of SARS-CoV-2 by qRT-PCR Technique

Paired nasopharyngeal and saliva swab samples were collected from 16 patients infected by SARS-CoV-2. Samples were classified as positive for SARS-CoV-2 when both the N1 and N2 protein primer-probe sets were detected. For the presence of the SARS-CoV-2 virus, specific probes for the detection of the virus in conjunction with the real-time reverse transcriptase polymerase chain reaction technique (qRT-PCR) were utilized.

### 4.3. Healthy Subject (Control Group)

Twenty healthy subjects (HS) were matched by age and gender. HS were negative for SARS-CoV-2. The collection of peripheral blood samples was carried out by venopunction. In these subjects, there was no suspicion of inflammatory disease or presence of degenerative disorders such as thyroid and autoimmune diseases, diabetes mellitus, dyslipidemia, and arterial hypertension. The intake of some medications that could interfere with the results of the study as antioxidants drugs and NSAIDs was considered, and the drugs were suspended 48 h before the obtainment of the sample. Control subjects reported not having any disease. Biochemical variables were also determined in these subjects such as glucose, insulin, cholesterol, triglycerides, HDL, LDL, and creatinine levels, to confirm that they really did not have alterations that could modify the results.

### 4.4. Therapeutic Management

The treatment applied during hospitalization was chosen according to standard maneuvers considering the requirements of each individual patient, the hemodynamic and electrolyte demands, and the ventilator demands. Treatment was initiated during the first hour after admission and before the recognition of the presence or absence of septic shock. Cultures were performed on admission before starting the administration of a broad-spectrum intravenous antibiotic.

Management with crystalloid solutions and/or albumin was considered depending on the hemodynamic status, by means of dynamic indicators. Use of vasopressors to maintain a media arterial pressure (MAP) ≥65 mmHg was given if necessary. Norepinephrine (NE) was the first option and/or vasopressin was used when there was a need to increase the MAP or reduce the NE dose. Inotropic drugs were administered in cases of myocardial dysfunction (dobutamine). Transfusion of blood packs were applied in case of a decrease in hemoglobin (<7.0 g/dL) in the absence of myocardial ischemia, severe hypoxemia, or severe bleeding. Mechanical ventilation with tidal volumes of 6 mL/kg was used in ARDS patients [50]. Plateau pressure was maintained at ≤30 cm H_2_ O, and alveolar conduction pressure of ≤13 cm H_2_ O. Positive end expiration pressure titration was managed by the use of the FiO_2_/PEEP (fraction inspired of oxygen/positive end expiration pressure). The treatment with anticoagulants was based on the Thachil guidelines [51]. The management with the prone position was necessary in patients with PaO_2_/FiO_2_ of ≤150 [52]. In all patients, the standard therapeutic management with dexamethasone 8 mg i.v., every 24 h for 7 days was applied between day 1 and 21 of the onset of symptoms when not contraindicated, and pentoxifylline tablets of 400 mg every 12 h by oral route or nasal-enteral tube for 5 days [53]. It was counter indicated when there was a requirement of O_2_ > 3 L, progressive requirement of =2, PAO_2_/FiO_2_ ≤250 mmHg, O_2_ use plus bilateral infiltrates in the radiography, O_2_ use plus DHL ≤250 U/L or ferritin ≥300 or DD ≥1000 ng/mL, CPK ≥2 times the upper normal value. The following conditions were not considered as counter indications or relative counter indications: glucose >250 mg/dL with hypoglycemic, hypokalemia <3.3 meq, blood pressure >155/95 mmHg with antihypertensive treatment, glaucoma, triglycerides >500 mg/dL (start treatment), history of known peptic ulcer or bleeding from recent gastrointestinal tract, untreated or decompensated dementia or psychiatric illness, use of non-potassium sparing diuretics or use of inhaled B2 agonists. The next conditions were monitored at follow-up: pre-prandial capillary glucometer (7–13–19 hrs.) for 10 days, even in fasting patients, MAP per shift and basal potassium every 72 h.

### 4.5. Doses of the NAC Therapy

Effervescent tablets of NAC 600 mg were used every 12 h by the oral route or nasal-enteral tube for 5 days. To administer the NAC therapy, a medical management algorithm was used considering the presence of comorbidities in each patient [3]. The NAC was adjusted to each comorbid condition or to the presence of potential allergies or heart rhythm disorders due to each individual history. All data entry was monitored at the coordinating center, with site visits for source data verification.

### 4.6. Blood Sample Obtainment and Storage

Blood samples were obtained from each patient that entered the hospital. The blood samples of both COVID-19 patients and HS were centrifuged for 20 min at 936 g and 4 °C. The serum of the samples was placed in aliquots and stored at −30 °C. Laboratory tests were carried out for the COVID-19 patients to determine the acute-phase reactants, urea nitrogen, creatinine, glucose, hemoglobin, leukocytes, lymphocytes, platelets, albumin, D-dimer, fibrinogen, ferritin, C-reactive protein, procalcitonin, and interleukin-6 (IL-6). Data from the patient’s medical history including demographic, prior illnesses to SARS-CoV-2 infection, COVID-19 test result, whether mechanical ventilation was used, and treatment type given were used for the results analysis. Additionally, other biochemical variables were determined in serum from the patients infected by SARS-CoV-2 and HS such as OS markers and antioxidant enzymatic system.

### 4.7. Oxidative Stress Markers

#### Evaluation of Total Antioxidant Capacity (TAC)

In total, 100 μL of serum was suspended in 1.5 mL of a reaction mixture prepared as follows: 300 mM acetate buffer pH 3.6, 20 mM FeCl_3_ 6 H_2_ O, and 10 mM of 2,4,6-tris-2- pyridyl-s-triazine were dissolved in 40 mM HCl. These reactants were added in a ratio of 10:1:1 *v*/*v*, respectively. After mixing, the samples were incubated at 37 °C for 15 min in the dark. The absorbance was measured at 593 nm [15].

### 4.8. Glutathione Levels (GSH)

This test determines the oxidation of GSH by means of Ellman’s reagent to form a yellow chromophore called 5′-thio-2-nitrobenzoic (TNB) and is quantified by a spectrophotometer. The rate of formation of TNB is proportional to the concentration of GSH in the sample. About 100 μL of serum was used according to a previously described method by Ellman. The calibration curve was obtained with solution GSH 1 mg/1 mL and the absorbance was measured at 412 nm [15].

#### 4.8.1. Determination of Total Thiol Groups

This test consists of the Ellman’s reagent reaction with a thiol group, commonly a thiolate, producing thoil-nitrobenzoate through potassium hydride. The technique used was previously described by Erel and Neselioglu [53], with some modifications carried out in our laboratory as previously reported [15]. About 50 μL of serum was used for the determination and the absorbance was measured at 415 nm. The calibration curve was obtained with solution GSSG 1 mg/1 mL and the absorbance was measured at 415 nm.

#### 4.8.2. Determination of Lipid Peroxidation (LPO) Levels, Aldehyde Deshydrogenase Activity (ALDH) and 3-Nitrotyrosine (3-NT)

This test is based on the malondialdehyde reaction, a secondary product of the oxidation of fatty acids with three or more bonds, with thiobarbituric acid in an acid medium and at high temperature, generating a pink-colored product. A total of 100 μL of serum was used for this determination and the absorbance was measured at 532 nm [52]. The activity of ALDH was determined through the colorimetric assay kit provided by Sigma-Aldrich (MAK082-1 KT), and 50 μL of serum was used for the determination. The 3-nitrotyrosine (3-NT) determination was carried out with a kit provided by LifeSpan BioSciencies, Seattle, WA, USA kit No. LS-40120). This assay is based on the competitive ELISA principle and is measured at a wavelength of 450 nm, using a visible light micro plate reader (Stat Fax 3200 Awareness Technology, Palm City, FL, USA), and 25 μL of serum was used for this determination.

#### 4.8.3. Nitrites (NO_2_^–^)

The NO_2_^–^ levels in serum were determined by the Griess reaction and a total of 100 µL of serum was used. NO_2_^−^ reacts in acid media with sulfanilic acid to form a diazonium cation which is then coupled to the aromatic amine 1-naphthylamine to produce a red-violet coloration that can be read on the spectrophotometer at 540 nm.

### 4.9. Determinations of TrxR, GPx, GST and GR Activities

The TrxR activity was determined using 50 μL of serum according to the method described by Pérez Torres et al. [54]. The sample was incubated and monitored at 412 nm for 6 min at 37 °C. To evaluate the GPx, GST, and GR activities, 50 μL of serum was used as was previously described [54]. The samples were incubated and monitored at 340 nm for 6 min at 37 °C.

The activity of extracellular super oxide dismutase (EcSOD) was determined in serum through non-denaturing gel electrophoresis according to the method previously report by Rodríguez-Fierros et al. [55]. A total of 25 μL of serum was applied directly in non-denaturing 10% polyacrylamide gels. The electrophoresis was carried out at 120 volts for 4 h. Subsequently, the gel was incubated with 2.45 mM nitro blue tetrazolium (NBT) solution for 20 min, then incubated with 28 mM EDTA solution, containing 36 mM potassium phosphate (pH 7.8) and 0.028 mM riboflavin, and exposed 10 min by UV light. Purified SOD from bovine erythrocytes with a specific activity of 112 U/mg of protein (Sigma-Aldrich, St. Louis, MO, USA) was used as a positive control for calculating the activity of the enzyme.

### 4.10. Interleukin-6 Concentration

IL-6 levels were measured in plasma samples by an enzyme-linked immunosorbent assay (ELISA) using a commercial kit according to the manufacturer’s instructions (BioLegend, San Diego, CA, USA), and 50 μL of serum was used for this determination.

### 4.11. Statistical Analysis

Continuous variables were expressed as median, first quartile, second quartile, interquartile range with minimum and maximum range. Categorical variables were expressed as frequencies and percentages. SigmaPlot^®^ version 14.5. (Systat Software Inc., San Jose, CA 95131, USA, EE.UU, North First Street, Suite 360, Jandel Corporation, San Jose, CA, USA) was used to generate the analysis and graphs. Continuous variables were compared with the Mann–Whitney U rank sum test followed by the normality test (Shapiro–Wilk) between HS vs. before the NAC treatment SARS-CoV-2 infection and by Kruskall–Wallis test before the NAC treatment SARS-CoV-2 infection vs. after the NAC treatment SARS-CoV-2 infection. Differences were considered statistically significant when *p* ≤ 0.05.

## 5. Conclusions

The treatment with NAC as an adjuvant therapy may contribute to reducing the OS associated with SARS-CoV-2 infection by restoring the loss of the redox homeostasis through the generation of GSH. Moreover, GSH promotes the metabolic pathways that depend on it, contributing to an increase in the TAC and to restore the redox homeostasis.

## Figures and Tables

**Table 1:** Demographic characteristics at admission in patients infected with SARS-CoV-2 before the NAC treatment.

Variables	Median and (Min–Max)
Age	55 (27–84)
BMI	28 (23–38)
Comorbidities	N (%)
Diabetes Mellitus	8 (47)
Hypertension	8 (47)
Dyslipidemia	7 (41)
Normal Weight	6 (35)
Overweight	7 (41)
Obesity	3 (24)
Signs	Median (Min–Max)
PaO_2_	70 (59–179)
PCO_2_	31.7 (12–39)
PaO_2_/FIO_2_ (mmHg)	131.5 (45–282)
SPO_2_/FIO_2_ (mmHg)	119 (61–290)
HR lpm	74 (20–116)
MAP (mmHg)	78 (71–102)
Temperature °C	36 (36.5–37.2)
Glucose	141 (100–359)
Creatinine in serum mg/dL	0.85 (0.50–1.30)
BUN	17 (5.6–36.6)
TC mg/dL	133 (89–210)
TG mg/dL	186.5 (80–726)
HDL-Cholesterol mg/dL	26 (14–40)
LDL mg/dL	62 (40–1109)
Leukocytes 10^^3^/µL	8.8 (3.5–14.9)
Lymphocytes 10^^3^/µL	8.3 (0.14–1.54)
Platelets 10^^3^/µL	253 (164–408)
Ferritin ng/mL	435.4 (146–2592)
D-dimer ng/dL	605 (200–2100)
IL-6 pg/mL	16.2 (7.8–324)
N/L	8.4 (3–67)
C reactive protein mg/dL	143.15 (44.7–221)
Procalcitonin ng/dL	0.23 (0.08–11.4)
SOFA	0 (0–3)
APACHE	5.5 (4–8)
SAPS	27 (18–32)
Patients in moderate condition	8 (47%)
Patients with severe condition	9 (53%)
Days With Mechanic ventilation	13 (5–28)
Days in ICU	17 (3–30)

Abbreviations: BMI = body mass index, HR = heart rate, MAP = mean arterial pressure, HDL = high-density lipoproteins, LDL = low-density lipoproteins, IL = interleukin, N/L = neutrophils and/or lymphocytes. FiO2 = fraction of inspired oxygen, PAO2 = oxygen at arterial pressure, PCO2 = carbon dioxide at partial pressure, SpO2 = arterial oxygen saturation, TG = triglycerides. SOFA = Organ Failure Sequential Assessment, APACHE = Acute Physiology and Chronic Health Assessment II, SAPS = Simplified Acute Physiology II Score.

**Table 2:** Gender, age, and serum biochemical determinations in the healthy subjects.

Variable	Median and (Min–Max)
Gender	10 men and 10 female
Age	53 (30–63)
Glucose (mg/dL)	83.0 (62.0–120.0)
Insulin (ng/mL)	0.3 (0.15–0.47)
Cholesterol (mg/dL)	169.0 (143.0–209.0)
Triglycerides (mg/dL)	93.5 (45.0–223.0)
HDL (mg/dL)	41.5 (32.0–80.0)
LDL (mg/dL)	94.5 (65.0–121.0)
Creatinine (mg/dL)	0.8 (0.69–0.9)

The results are shown median and min and max range.

**Table 3:** Oxidative stress markers in healthy subjects and patients infected with SARS-CoV-2.

Variables (mL/Serum)	HS Median (Min–Max) (Q1, Q3, IQR)	Before the NAC Treatment SARS-CoV-2 Infection Median and (Min–Max) (Q1, Q3, IQR)	After the NAC Treatment SARS-CoV-2 Infection Median and (Min–Max) (Q1, Q3, IQR)
TAC (nM Trolox)	12,783.5 (8111.7–33,063.9) (9598.1, 26,719.6, 17,121.4)	5727.1 (4710.5–7034.6) *** (5143.2, 6527.7, 1384.4)	7143.3 (6193.6–10,021.6) *** (6564.9, 7730.4, 1165.5)
GSH (µM)	0.07 (0.05–0.22)	0.03 (0.01–0.07) ***	0.07 (0.03–0.43) ***
	(0.06, 0.10, 0.03)	(0.03, 0.04, 0.01)	(0.04, 0.27, 0.23)
Thiols (µM)	9.1 (3.3–16.4)	3.1 (0.4–6.6) ***	4.7 (0.7–9.8)*
	(6.4, 11.1, 4.6)	(1.6, 4.5, 2.9)	(3.1, 6.8, 3.6)
LPO (nM MDA)	1.2 (0.3–2.7)	2.1 (0.6–3.6) **	1.1 (0.2–2.6) *
	(0.7, 1.8, 1.1)	(1.2, 2.7, 1.5)	(0.3, 2.3, 1.9)
NO_2_ (nM)	22.0 (10.7–67.5)	3.5 (2.5–11.1) ***	24.1 (9.5–45.7) ***
	(19.9, 28.6, 8.7)	(2.9, 6.5, 3.6)	(15.0, 30.7, 15.7)
3-NT (nM)	7.0 × 10^−7^ (1.3 × 10^−8^–8.0 × 10^−3^) (4.4 × 10^−7^, 5.6 × 10^−3^, 5.6 × 10^−3^)	7.1 × 10^−3^ (5.3 × 10^−4^ – 2.1 × 10^−2^) *** (2.9 × 10^−3^, 9.8 × 10^−3^, 6.9 × 10^−3^)	1.2 × 10^−2^ (2.8 × 10^−3^–4.4 × 10^−2^) * (6.2 × 10^−3^, 1.6 × 10^−2^, 1.0 × 10^−2^)

*** *p* < 0.001, ** *p* = 0.01, significantly changes between HS vs. before of the NAC treatment SARS-CoV-2 infection and *** *p* < 0.001, * *p* = 0.02, * *p* ≤ 0.05 before of the NAC treatment SARS-CoV-2 infection vs. after of the NAC treatment SARS-CoV-2 infection. Abbreviations: HS = healthy subjects, TAC = total antioxidant capacity, GSH = glutathione reduced, LPO = lipid peroxidation, NO_2_^–^ = nitrite, 3-NT = 3-nitrotyrosine. Q1 = first quartile, Q2 = second quartile, IQR = interquartile range. The results are shown median, first quartile, second quartile, interquartile range and min and max range.

## Data Availability

The datasets generated and analyzed during the current study are available from the corresponding author on reasonable request.
